# Soy Protein Containing Isoflavones Improves Facial Signs of Photoaging and Skin Hydration in Postmenopausal Women: Results of a Prospective Randomized Double-Blind Controlled Trial

**DOI:** 10.3390/nu15194113

**Published:** 2023-09-23

**Authors:** Julianne Rizzo, Mildred Min, Sarah Adnan, Nasima Afzal, Jessica Maloh, Cindy J. Chambers, Vivien Fam, Raja K. Sivamani

**Affiliations:** 1 Integrative Skin Science and Research, Sacramento, CA 95815, USA; 2 School of Medicine, University of California-Davis, Sacramento, CA 95817, USA; 3 Zen Dermatology, Sacramento, CA 95819, USA; 4 Pacific Skin Institute, Sacramento, CA 95815, USA; 5 College of Medicine, California Northstate University, Elk Grove, CA 95757, USA; 6 Department of Dermatology, University of California-Davis, Sacramento, CA 95616, USA

**Keywords:** soy, isoflavones, skin aging, photoaging, wrinkles, hydration, soy protein

## Abstract

Preliminary findings from multiple studies indicate that dietary intake of soy-derived isoflavones exert beneficial effects on the skin including defense against oxidant damage, stimulation of collagen synthesis, and increased hydration. This study aims to investigate how oral supplementation of a soy protein isolate with added isoflavones (SPII) affects components of photoaging such as facial wrinkles and dyspigmentation, and skin biophysical measures such as skin hydration and sebum excretion in postmenopausal women. This 6-month prospective, randomized double-blind controlled study was conducted on 44 postmenopausal women with Fitzpatrick skin types I, II, and III who were randomized to receive either casein protein or SPII. A high-resolution facial photography system was used to measure wrinkle severity and pigmentation at 0, 8, 16, and 24 weeks. Skin biophysical measurements included skin hydration and sebum production. The average wrinkle severity was decreased in the SPII intervention group at week 16 and week 24 by 5.9% and 7.1%, respectively, compared to the baseline. Compared to the casein group, average wrinkle severity was significantly decreased at week 16 (*p* < 0.05) and week 24 (*p* < 0.0001). Facial pigment intensity was decreased by −2.5% (*p* < 0.05) at week 24, whereas there was no significant change in the casein group. Compared to baseline, skin hydration in the SPII group was significantly increased by 39% and 68% on the left and right cheeks (*p* < 0.05), respectively, at 24 weeks. There were no significant differences in sebum production. Dietary soy protein supplementation with isoflavones may improve skin photoaging, including wrinkles and dyspigmentation, and increase skin hydration in postmenopausal women with Fitzpatrick skin types I, II, and III.

## 1. Introduction

Plant-derived isoflavones are naturally occurring nonsteroidal compounds that resemble estrogens and are commonly classified as phytoestrogens [1]. Due to their structural similarity to estradiol, isoflavones are able to bind to estrogen receptors and, under some experimental conditions, mimic their actions [2]. Specifically, isoflavones can bind to ERα and ERβ receptors, which have been shown to have chemoprotective effects and interact with keratinocytes and fibroblasts in the skin [3]. ERβ activation has also been shown to encourage wound healing [4]. As a result, isoflavones may promote cell proliferation [5], act as antioxidants [6], and exert anti-inflammatory and anti-cancer properties [7,8]. In the skin, isoflavones and other phytoestrogens have also been shown to increase hyaluronic acid concentration [9], the content and quality of collagen [10], and stimulate the synthesis of extracellular matrix proteins [11]. Thus, isoflavones have become a growing topic of interest in dermatology.

The main dietary sources of isoflavones for humans are soybean and soy-derived products, which primarily contain daidzein and genistein [12]. One pilot study showed that the use of an isoflavone-rich soy extract for six months in postmenopausal women significantly increased epithelial thickness, elasticity, collagen fiber quantity, and angiogenesis [13]. In rats, soy isoflavones significantly increased the thickness of the skin epidermis, collagen fibers in the dermis, and the number of elastic fibers [14]. Soy isoflavones also contain serine protease inhibitors that mediate protease-activated receptor-2-mediated phagocytosis of melanosomes by keratinocytes, thereby minimizing melanin pigment transfer to surrounding keratinocytes in the skin [15]. 

Isoflavones may modulate women’s health through multiple modalities. Supplementation has been shown to mitigate multiple symptoms of perimenopausal and postmenopausal women [16,17]. Some of the benefits include reduction in estrogen sensitive cancer risks, reduce bone mineral density loss, and improve glycemic control [18,19,20]. Isoflavone supplementation has also been shown to be beneficial for acne vulgaris and reduced circulating levels of dihydrotestosterone (DHT) [21]. Taken together, soy isoflavones are supporting of hormonal shifts in women whether they are in the premenopausal, perimenopausal, or the post-menopausal state.

Postmenopausal women are more susceptible to facial aging due to a shift in hormonal balance and decrease in collagen synthesis [22]. Although skin aging is a multi-factorial process, there is increasing evidence that nutrition and dietary habits can modulate oxidative stress and inflammation, which may accelerate this process [23]. Fitzpatrick skin type also plays a role in skin aging. Fitzpatrick skin phototyping classifies skin to types 1 to 6 based on an individual’s tendency to burn versus tan after sun exposure [24]. Generally, the higher the skin type, the more likely the skin tans more easily than burns, and a higher level of melanin will be present in the skin [25]. Thus, skin types 1, 2, and 3, which are more sensitive to ultraviolet light and burn more easily than tan, may experience accelerated photoaging [26].

Food-based interventions have previously demonstrated modulatory effects on skin aging [27]. For example, the consumption of carotenoid-rich foods has been shown to have photoprotective effects. One study found that a tomato nutrient complex containing lycopene and lutein inhibited ultraviolet-induced upregulation of molecules indicated in oxidative stress and photoaging [28]. Another study found that 85 g of Ataulfo mangos, which are rich in beta-carotene and other carotenoids, significantly reduced wrinkle severity in postmenopausal women [29]. Additionally, carotenoid-rich foods exert pro-vitamin A activity which results in modulation of cellular differentiation and increased epidermal proliferation to decelerate photoaging [30].

Foods rich in antioxidants such as vitamin E (alpha-tocopherol) have also been implicated in facial wrinkles and pigmentation. Almonds are one example of a food that contains alpha-tocopherol. Two studies demonstrated that daily consumption of almonds in postmenopausal women with Fitzpatrick skin types 1–3 for either 16 or 24 weeks resulted in a significant decrease in wrinkle severity and facial pigment intensity [31,32]. Topically, naturally occurring vitamins in foods, such as alpha-tocopherol, have been seen to reduce dark under-eye circles and eye wrinkles [33]. As increasing evidence supports the use of food supplementation for skin aging, the food-based cosmetic industry continues to grow. By the end of 2023, this market is estimated to reach a market value of USD 6.8 billion [34]. 

Soy isoflavones have garnered interest for a range of functions including their antioxidant, phytoestrogenic, and anti-inflammatory properties [35,36,37]. In cell culture and animal studies, soy isoflavones have been shown to protect against ultraviolet type B-related cells death, erythema, skin barrier dysfunction, and depletion of the skin’s natural antioxidant enzyme catalase [38]. Soy isoflavones also reduce skin hyperpigmentation through a reduction in melanin transfer from melanocytes to keratinocytes [39]. Furthermore, soy-derived protein and peptides have been studied for their impact on collagen synthesis. Soy-derived peptides were shown to be similar to collagen-derived peptides in stimulating collagen synthesis in dermal fibroblasts [40]. Another study showed that the ingestion of soybean extracts increased the thickness of collagen in rats [41]. 

As a result of these findings with soy isoflavones and soy protein, there has been growing interest to assess the intake and supplementation of soy-based products for skin health. A previous study assessing postmenopausal women with Fitzpatrick skin types 1–3 who were supplemented with a mixture of soy isoflavones, lycopene, vitamin C, vitamin E, and fish oil for 14 weeks showed a reduction in wrinkle depth [42]. 

The current study builds on all of these preliminary studies to comparatively study soy protein containing isoflavones (SPII) against a calorie-matched casein protein without added isoflavones. Specifically, this study investigates how supplementation with SPII shifts skin health including the appearances of wrinkles and pigmentation as well as skin biomechanical properties such as hydration and sebum production. 

## 2. Methods and Materials

### 2.1. Materials

The SPII used in this study was provided by Dupont (Wilmington, DE, USA) and contained the following ingredients: soy protein (isolated soy protein with less than 2% lecithin), sugar, fructose, resistant maltodextrin, spray-dried corn oil shortening (corn oil, corn syrup solids, sodium caseinate, and BHT and propyl gallate (to help protect flavor)), natural and artificial flavor, xanthan gum, salt, vitamin/mineral (sodium ascorbate, maltodextrin, vitamin E, ferric orthophosphate, niacin, calcium pantothenate, zinc oxide, manganese sulfate, pyridoxine hydrochloride, riboflavin, thiamin, vitamin A, chromium chloride, folic acid, biotin, potassium iodide, sodium molybdate, sodium selenite, vitamin K, vitamin D, and vitamin B12), sucralose, acesulfame k, and powder. The total caloric content for 46 g of the powdered SPII product was 170 calories, with a breakdown of 2 g of total fat (3% of daily value), 550 mg of sodium (24% of daily value), 10 g of total carbohydrates (4% of daily value), 3 g of added sugars (6% of daily value), and 30 g of protein.

The casein protein powder that served as a control consisted of the following: sodium caseinate, sugar, fructose, resistant maltodextrin, spray dried corn oil shortening (corn oil, corn syrup solids, sodium caseinate, and BHT and propyl gallate (to help protect flavor)), natural and artificial flavor, xanthan gum, salt, vitamin/mineral (sodium ascorbate, maltodextrin, vitamin E, ferrice orthophosphate, niacin, calcium pantothenate, zinc oxide, manganese sulfate, pyridoxine hydrochloride, riboflavin, thiamin, vitamin A, chromium chloride, folic acid, biotin, potassium iodide, sodium molybdate, sodium selenite, vitamin K, vitamin D, and vitamin B12.), sucralose, acesulfame k, and powder. The total caloric content for 45 g of this powered casein powder was 170 calories, with a breakdown of 1.5 g of total fat (2% of daily value), 430 mg of sodium (19% of daily value), 10 g of total carbohydrates (4% of daily value), 3 g of added sugars (6% of daily value), and 30 g of protein.

### 2.2. Study Design, Recruitment, and Randomization

This prospective, double-blind, randomized controlled trial was conducted in the greater Sacramento area at Integrative Skin Science and Research (Sacramento, CA, USA). Methods of recruitment included local dermatology clinics and social media advertising. The study protocol and consent were reviewed and approved by the Allendale Institutional Review Board (Protocol ID: SYW_01) and registered at www.clinicaltrials.gov (NCT04871750). Enrolled study participants were randomized to either 30 g/d of soy protein containing 50 mg isoflavones or 30 g/d of casein for a total of 24 weeks. Subjects, clinical research coordinators, and those assessing outcomes were all blinded to the intervention. Study visits occurred at screening, baseline, 8, 16, and 24 weeks. Facial photography was taken, and skin biophysical measurements were recorded at all visits except screening. 

**Figure 1:**
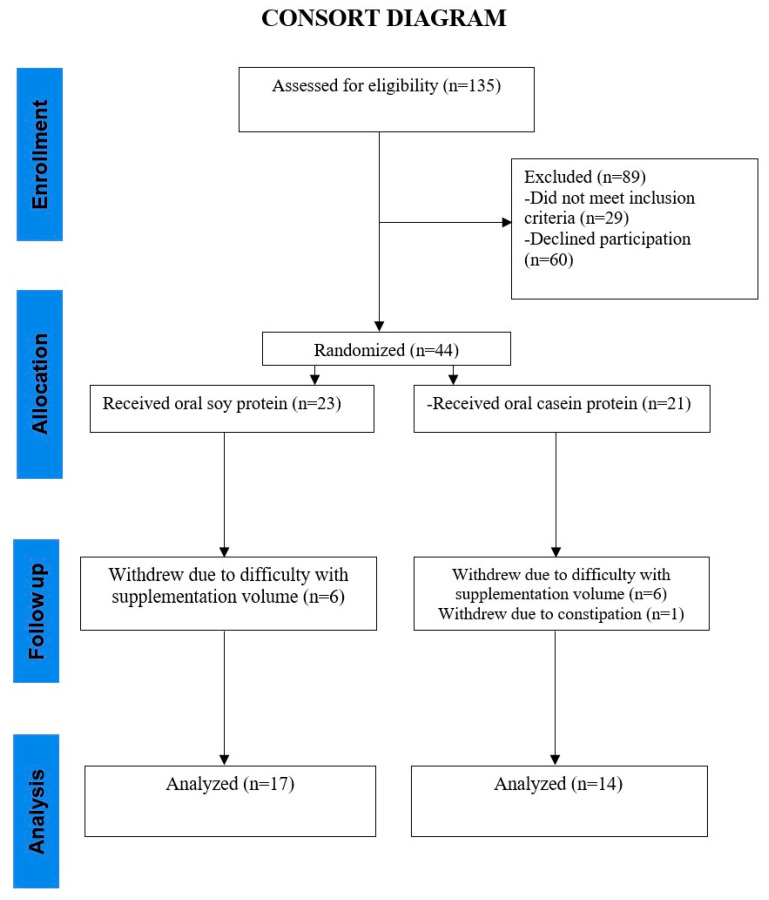
CONSORT (Consolidated Standards of Reporting Trials) flow diagram.

In total, 135 individuals were assessed for eligibility and 44 participants were randomized to either SPII or casein. A CONSORT diagram is presented in Figure 1. Any adverse events of the intervention were noted throughout the study.

### 2.3. Inclusion and Exclusion Criteria

Healthy postmenopausal women aged 50 to 70 were eligible to participate if they were Fitzpatrick skin types I, II, or III and had a BMI between 18.5 and 35 kg/m^2^. Participants agreed to maintain their current diet for the duration of the study. Exclusion criteria included those on any hormone replacement therapy, systemic antibiotics within six months of enrollment, topical antibiotic or benzoyl peroxide use within one month of enrollment, allergies to soy and milk products, fruit consumption > 2 cups/d, vegetable consumption > 3 cups/d, fruit juice consumption > 1 cup/d, nut consumption > 2 oz/d, coffee consumption > 3 cups/d, alcohol consumption > 3 servings/week, self-reported malabsorption, current cancer or kidney, liver, or gastrointestinal disease, oral probiotic or prebiotic supplementation within 1 month of enrollment, commencement of a new diet within one month of enrollment, use of medications that alter blood lipids such as statins, current tobacco smokers, and those unable to consent.

### 2.4. Facial Imaging and Skin Biophysical Measurements

The BTBP 3D Clarity Pro^®^ Facial Modeling and Analysis System (Brigh-Tex BioPhotonics, San Jose, CA, USA) was utilized to obtain high-resolution facial photographs for all study participants at baseline and at 8, 16, and 24 weeks. Facial wrinkles were analyzed through the BTBP 3D Clarity Pro^®^ Facial Modeling and Analysis System (Brigh-Tex BioPhotonics), which uses an algorithm-based model to measure and calculate the average wrinkle severity based on the depth and width [43]. Average piment intensity score across the face was also measured utilizing the same image analysis system. The following skin biophysical measures were assessed: hydration (MoistureMeterSC; Delfin Technologies, Stamford, CT, USA) and sebum production in µg/cm^2^ (Sebumeter^®^ SM 815; Courage and Khazaka, Cologne, Germany). Skin hydration (MoistureMeterSC; Delfin Technologies) and sebum excretion (Sebumeter^®^ SM 815; Courage and Khazaka) were measured utilizing handheld non-invasive devices at the left and right buccal areas at 0, 8, 16, and 24 weeks. All subjects were acclimated to ambient temperature for fifteen minutes in a temperature-controlled room prior to any skin biophysical measurements. Subjects were instructed to refrain from applying topical products prior to study visits.

### 2.5. Statistical Analysis

The primary endpoint of this study was to assess whether SPII supplementation could decrease wrinkle severity and pigment intensity compared to the casein group after 24 weeks. Secondary endpoints included assessments of skin hydration and sebum excretion within groups. The data were analyzed at 0, 8, 16, and 24 weeks. Statistical comparisons were made by using a Student’s *t*-test for within group (paired, two-tailed) and between group (unpaired, two-tailed) differences. Statistical significance was set at *p* ≤ 0.05. The results are presented as the mean with standard error of mean. Baseline data for subjects were utilized as controls for within-group comparisons. Prism v.10 (GraphPad Software LLC, San Diego, CA, USA) was used to analyze and visualize the data.

## 3. Results

Out of 135 participants who were screened, 44 postmenopausal females met the enrollment criteria and were randomized into the SPII (n = 23) or the casein group (n = 21). The mean (SD) age for participants in SPII and casein groups was 62 ± 6 y and 64 ± 4 y, respectively.

### 3.1. Imaging System-Based Photographic Analysis of Wrinkle Severity and Pigment Intensity

**Figure 2:**
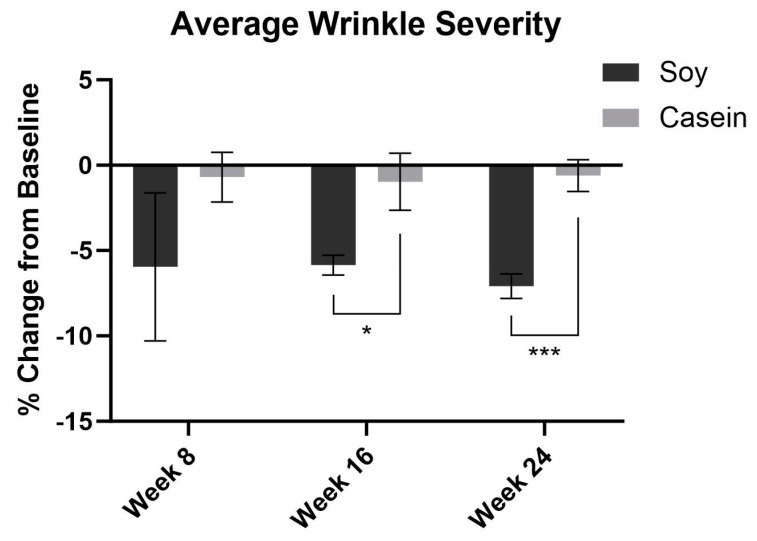
Computer-based photographic analysis of wrinkle severity was significantly decreased in the soy protein intervention group compared to the casein protein intervention group by −4.8% and −6.5% at week 16 and 24, respectively. Error bars represent the standard error of the mean (SEM). * = *p* < 0.05, *** = *p* < 0.0001.

**Figure 3:**
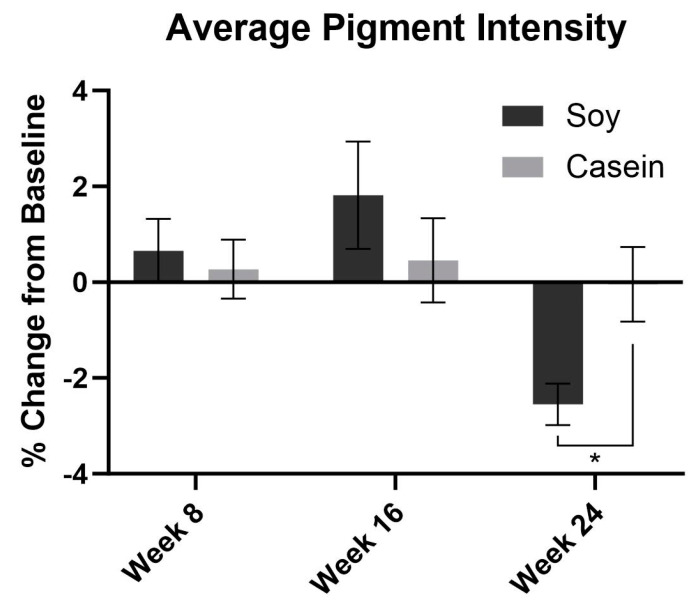
Computer-based photographic analysis of pigment intensity was significantly decreased in the soy protein intervention group compared to the casein protein intervention group by −2.5% at week 24. Error bars represent SEM. * = *p* < 0.05.

The average wrinkle severity in the SPII group decreased by 5.9% and 7.1% at week 16 and 24, respectively. Wrinkle severity was decreased in the SPII group compared to the casein protein group at week 16 (*p* < 0.05) and 24 (*p* < 0.0001) (Figure 2). The average pigment severity in the SPII group decreased by 2.4% at week 24. The average pigment intensity was significantly decreased in the SPII group compared to the casein group at week 24 (*p* < 0.05) (Figure 3).

### 3.2. Skin Hydration

**Figure 4:**
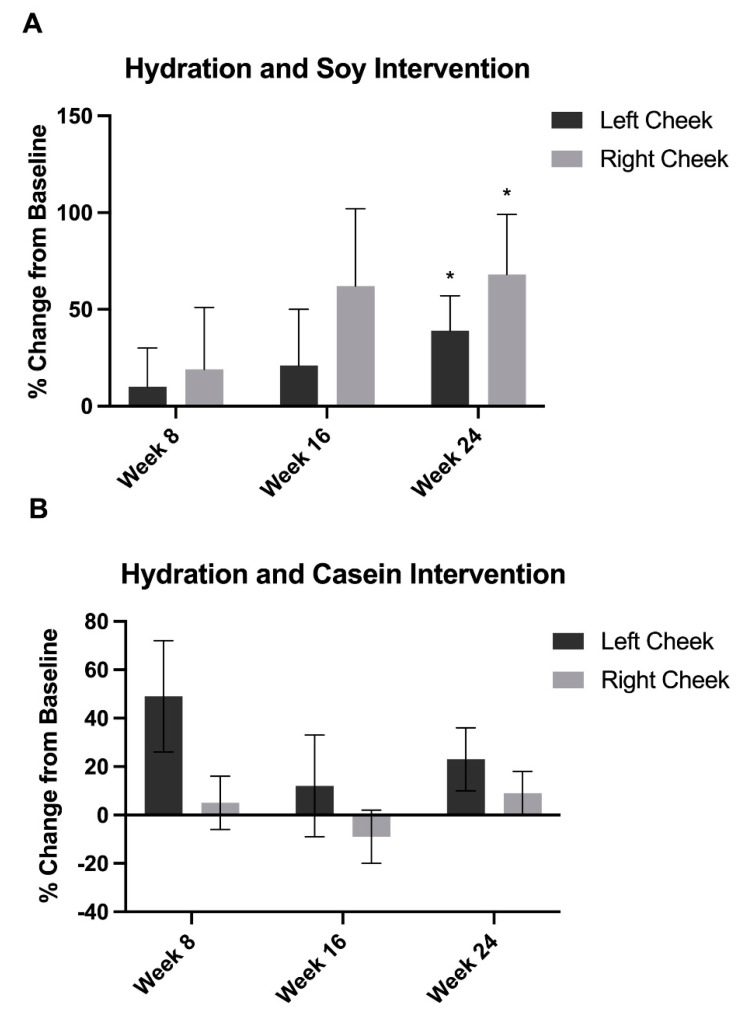
Skin hydration was measured resulting in a significant increase in hydration from baseline at week 24 in the (**A**) soy intervention group and no significant differences in hydration compared to baseline in the (**B**) casein intervention group. Error bars represent SEM. * = *p* < 0.05.

Skin hydration was significantly increased in the soy protein intervention group by 39% in the left cheek and 68% in the right cheek at week 24 (*p* < 0.05) compared to baseline (Figure 4). There were no statistically significant changes in the casein intervention group.

### 3.3. Sebum Excretion

**Figure 5:**
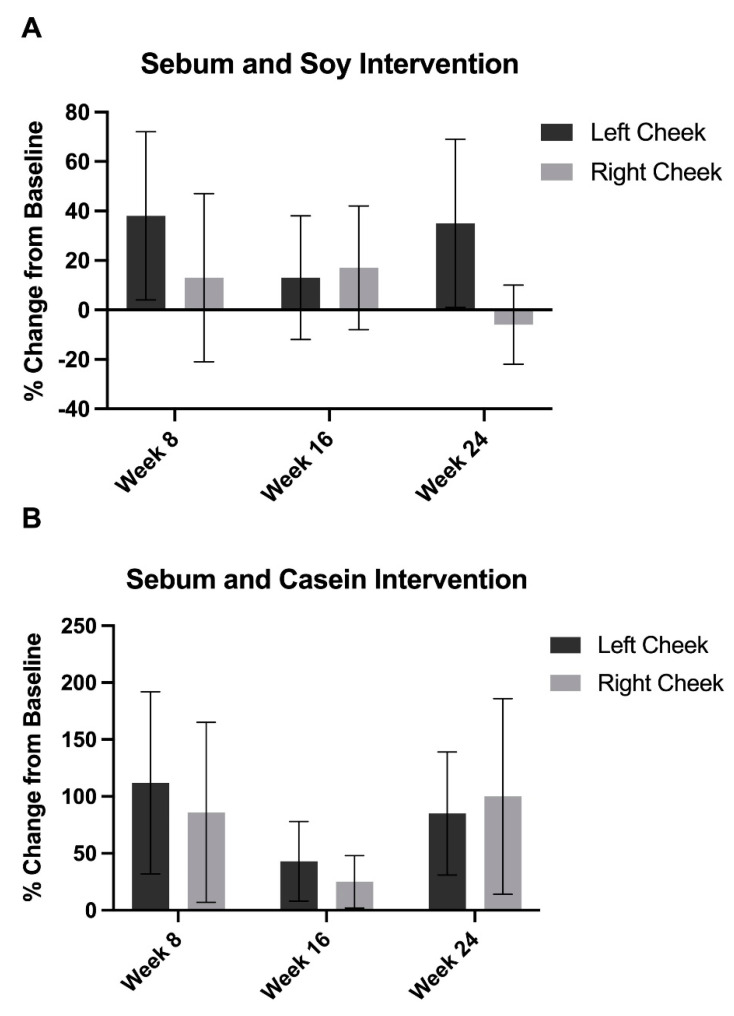
Sebum production did not change significantly in either the (**A**) soy intervention group or the (**B**) casein intervention group at week 8, 16, or 24 compared to baseline. Error bars represent SEM.

Compared to baseline, there were no significant changes in sebum production in either group at any time point (Figure 5).

### 3.4. Facial Photography

**Figure 6:**
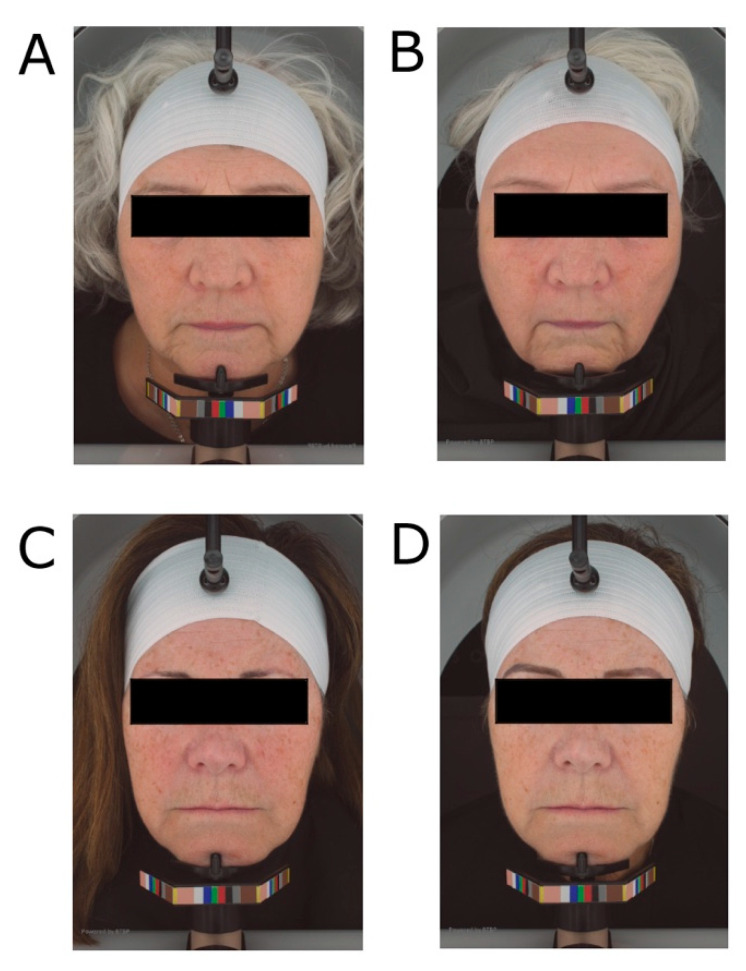
Soy protein isolate with added isoflavones subject at baseline (**A**) and 24 weeks (**B**). Casein supplemented subject at baseline (**C**) and 24 weeks (**D**).

High-resolution photography was taken for both groups at baseline and week 8, 16, and 24 (Figure 6).

### 3.5. Adverse Events

There was one episode of constipation that led to withdrawal in the casein group.

## 4. Discussion

In this 24-week, prospective, randomized controlled trial, 30 g/d of soy protein powder containing 50 mg of isoflavones daily showed significant improvements in wrinkle severity, pigmentation, and hydration at week 24. In addition to sun protective habits such as sunscreen use and sun protective clothing, the current results support the adjunctive use of soy-protein-derived isoflavones for the reduction in skin wrinkles and dyspigmentation such as occurs in skin photoaging. 

Our study supports the use of a soy-based protein supplement with isoflavones to improve signs of facial skin photoaging such as wrinkles and facial pigmentation in postmenopausal women with Fitzpatrick skin types 1–3. Compared to the casein protein group, the SPII group experienced a significant decrease in wrinkle severity at week 16 and 24, and significant decrease in average pigment intensity at week 24. The mechanism by which soy isoflavones mediate wrinkle severity and pigment intensity may be related to its antioxidative [44], collagen-promoting [42], and pigmenting evening properties [45]. Intra- and extracellular oxidative stress created by reactive oxygen species accelerate skin photoaging and dyspigmentation. Antioxidants scavenge reactive oxygen species that accelerate skin photoaging and dyspigmentation [46]. Soy isoflavones reduce oxidative stress through the inhibition of NF-кB [44,47], thereby producing antioxidative effects that correlate with the significant decreases in wrinkle severity and pigment intensity, as seen in this study. One study found that a dietary cocktail containing soy isoflavones increased collagen fiber synthesis in the dermis [42]. A strengthened collagen network would improve the appearance of fine lines and wrinkles. Additionally, soy-derived products reduce hyperpigmentation through serine protease inhibitors that inhibit keratinocytic phagocytosis of melanosomes [15,45]. Our findings are consistent with other studies that have shown that oral soy isoflavone intake improves signs of facial skin photoaging [48,49].

The finding that SPII supplementation lead to an improvement in the facial pigmentation has support from the literature. Soy-based extracts have been shown to reduce facial hyperpigmentation when applied topically [15]. The isoflavones appear to act through multiple mechanisms of action including inhibition of tyrosinase activity to reduction in melanogenesis and decreased responsiveness to alpha-MSH stimulation of melanocytes [50,51]. Our findings are not only statistically significant but they are clinically significant as well. The 5.9% decrease in wrinkle severity at week 16 and the 7.1% decrease in wrinkle severity at week 24 noted in this study are similar to the wrinkle severity decreases seen with other food-based supplementation studies. Almond supplementation in postmenopausal women was shown to decrease wrinkle severity by 9% at week 16 in a pilot study [32] and by 16% at week 24 in a follow-up study [31]. Mango supplementation in postmenopausal women showed a 20% decrease in wrinkle severity at week 16 [29] in the low dose supplementation group. With food-based interventions, it is not known how continued supplementation may further alter the wrinkle severity, but there was an increasing trend for continued improvement in this study from week 16 to week 24, suggesting that a future study over a longer time frame may be warranted.

The SPII group also experienced a significant increase in skin hydration at week 24 compared to baseline in both cheeks. Although this difference was not statistically significant, we chose to present the data as right and left cheeks rather than an overall average as there could be other confounders for variations in the cheeks such as driving-related sun exposure. For example, in the United States, where drivers experience increased ultraviolet (UV) exposure on the left side, malignant melanoma and merkel cell carcinoma were significantly more likely to occur on the left side of the face or arms [52]. One study found that when including all types of skin cancers and sexes, more skin cancers occurred on the left [53]. There have also been cases of unilateral dermatoheliosis [54] and unilateral solar purpura [55] as a result of asymmetrical photodamage in automobile drivers. Photodamage causes fragmentation of collagen and elastin, which decreases the hyaluronic acid content of the skin as the epidermis thins out, thereby reducing skin hydration [56]. Sleeping position can also contribute to the repetitive shear forces to facial skin that accelerate facial aging [57]. Additionally, there are variations in the distribution of eccrine glands, vasculature, and stratum corneum thickness across the cheek areas, which can also contribute to variable skin biophysical measurements from left to right cheeks [58]. Regardless, soy isoflavones have been shown to increase hyaluronic acid concentrations in the skin [9], and this may correlate with the increase in skin hydration that we noted in the SPII supplementation group. Hyaluronic acid is an important component of the extracellular matrix and acts as a biologic humectant to draw and retain moisture into the skin [59]. Thus, soy may increase the skin’s natural levels of hyaluronic acid, which would increase skin hydration and contribute to a reduction in the appearance of fine lines and wrinkles.

Estrogen-deficient skin, such as in postmenopausal women, is characterized by the loss of collagen, elastin, fibroblast function, and vascularity [4]. There is also increased cellular degradation which leads to dryness, atrophy, wrinkles, and decreased defense against oxidative stress [4]. All these changes contribute to the appearance of aging skin. As a result, phytoestrogens, which mimic estrogen’s effects on skin health, have become a major ingredient in skin care products for postmenopausal women [4]. Phytoestrogens act as selective estrogen receptor modulators (SERMs) and possess ERβ-agonist properties [60]. Upregulation of ERβ in dermal fibroblasts increases collagen synthesis and maturation [60]. Soy isoflavones exhibit phytoestrogenic effects that may influence collagen production and skin health and may be contributing to the results that we observed in this study.

Interestingly, neither intervention produced significant differences in sebum excretion. While the literature on the associations between soy and sebum production are lacking, one study found that the removal of precipitated casein in lactoferrin, a whey milk protein, led to decreased sebum production [61]. Future studies may elucidate trends associated with soy and sebum production.

As opposed to topical application, oral supplementation may have other benefits that could be considered an integrative and holistic approach to skin care. For example, soy protein supplementation may have an overall anti-inflammatory effect on the body with a reduction in the circulating IL-6 and TNF-alpha levels [62,63], although the impact on postmenopausal women remains unclear [64]. Furthermore, soy protein supplementation may improve bone health in post-menopausal women as well [65,66]. Finally, soy protein supplementation may diversify the gut microbiome [67]. Taken together, soy protein affects many aspects of health beyond the skin, making it a suitable part of an integrative approach to photoaging and health in postmenopausal women.

Our findings reveal several potential follow-up studies that would be warranted. For example, future studies should evaluate the role of SPII on other aspects of women’s health that can range from hair loss to acne to vaginal support. The role of supplementation-derived isoflavones vs. food-derived isoflavones should be explored further as well.

This study evaluated the use of soy protein containing 50 mg isoflavones and a previous study showed that consuming daily a soy extract containing 100 mg isoflavones improved epidermal thickness and increased collagen and elastin fibers in the dermis [13]. A similar level of isoflavones may be achieved through daily intake of soy-based foods such as tofu, soymilk, soy yogurt, edamame, and tempeh [68].

Although some concerns have arisen about the safety of isoflavones, particularly about the impact of soy on women with breast cancer. However, the evidence supports the opposite. Increasing consumption of soy isoflavones was associated with lowered risk for breast cancer in both pre-menopausal and post-menopausal women [69,70]. In fact, a systematic review that assessed the correlation between soy isoflavone intake and cancer in general found that increased soy isoflavone intake was associated with lowered cancer incidence [71]. The investigators calculated that there was a 4% reduction in the risk of cancer for each 10 mg/d increment of soy isoflavones that was consumed. Furthermore, a meta-analysis of prospective observational studies noted isoflavone intake reduced recurrence and breast cancer-specific mortality [72]. 

### Limitations

This study had numerous dietary restrictions that may not be fully representative of a person’s true diet outside of a clinical research setting. However, these restrictions also reduced potential confounding. This study had a relatively small sample size with all subjects being postmenopausal women, and the soy and casein were provided as protein powders that required the intake of large volumes of water on a daily basis and led to equal levels of withdrawals from each group. Nevertheless, our study was able to recruit enough participants to show statistically significant differences. Future studies should consider intervening with isoflavone-rich products that are less burdensome, such as traditional soy foods like tofu or isoflavone supplements. Future studies should also investigate how soy isoflavones in isolation may influence skin photoaging, whereas the SPII utilized in our study had a small proportion of added ingredients such as niacin and vitamin A, which may have contributed to dermatological effects of their own. This study was limited to Fitzpatrick skin types I, II, and III, and the results reported here cannot be generalized to all Fitzpatrick skin types. Similarly, because this study was limited to postmenopausal women, the results reported here cannot be extended to premenopausal or perimenopausal women. Because this study intervened with SPII with the active component of soy isoflavones, other isoflavone-rich soy foods such as tofu, soymilk, edamame, and tempeh may also exert similar benefits and should be the focus of future studies. 

## 5. Conclusions

In conclusion, supplementation with a standardized, multicomponent soy protein isolate with added isoflavones may improve facial wrinkle severity, reduce skin pigment intensity, and increase skin hydration in postmenopausal women with Fitzpatrick skin types I, II, and III. Future studies assessing supplementation of a soy protein with added isoflavones with an expanded population including males, younger participants, and higher Fitzpatrick skin types are warranted. Additionally, studies utilizing isolated soy protein and isoflavones to assess its effects on skin health may strengthen these associations. Since phytoestrogens may mimic estrogenic effects in the skin, future studies might also investigate the systemic effects of soy isoflavones in postmenopausal women from a gynecological or multidisciplinary perspective. 

## Data Availability

The data presented in this study are available on request from the corresponding author.
